# Acquired language disorders beyond aphasia: foreign accent syndrome as a neurological, speech, and psychiatric disorder

**DOI:** 10.3389/fpsyg.2025.1554104

**Published:** 2025-03-12

**Authors:** R. Stewart Longman, Flint D. Schwartz

**Affiliations:** ^1^Department of Psychology, University of Calgary, Calgary, AB, Canada; ^2^Alberta Health Services, Health Department in Grande Prairie, AB, Canada; ^3^Department of Psychology and Neuroscience, Dalhaousie University, Halifax, NS, Canada

**Keywords:** foreign accent syndrome (FAS), aphasia, history, Pierre Marie, imaging, Monrad-Krohn

## Abstract

This study examines historical conceptualizations of ‘foreign accent syndrome’ after brain trauma or as an aspect of psychiatric presentations, in addition to comparisons with current conceptualizations. Although classical understanding of aphasias as language disorders developed between 1861 and 1885, descriptions of non-aphasic speech disorders emerged later. Acquired accent following a stroke was first described in 1907 by Pierre Marie (1853–1940) in the context of the localizationist versus holistic debate. Early characterizations by Marie, Arnold Pick (1851–1924), and G.H. Monrad-Krohn (1884–1964) identified persisting speech changes following initial aphasia, which, from a contemporary viewpoint, provide insights into the dynamic nature of recovery after cerebral injury. These cases significantly contributed to the understanding of the neurological foundations of prosody and the non-linguistic aspects of speech. A deeper understanding of this disorder awaited contributions from various fields, including linguistics, speech-language pathology, psychiatry, and neuroimaging. Notably, there is an unusual gap in psychiatric causation reports prior to 1960, despite some intriguing indications from Josef Breuer’s account of Anna O (1895). This study explores how historical perspectives continue to influence current conceptualizations of foreign accent syndrome.

## Introduction

One of the triumphs of 19th-century neurology was the development of the concept of language localization, notably associated with Broca and significantly elaborated by Wernicke, along with conceptual maps such as Lichtheim’s “house” (Eling and Whitaker, 2022). Almost half a century later, neurologists began investigating changes in our speech, particularly the sudden emergence of “pseudo-accents,” now commonly referred to as “foreign accent syndrome.” This study reviews some of the history of these developments and, as far as we can determine, the surprisingly late inclusion of psychological or behavioral contributions to these conceptualizations. Initially, case descriptions supported specific models of aphasia, but they have since become intriguing in their own right, informing our understanding of the social effects of neurological presentations.

The term “foreign accent syndrome” was coined by Whitaker (1982), replacing various earlier terms such as anarthria, dysprosody, and aphemia. Whitaker defined foreign accent syndrome (FAS) as having four main characteristics: (1) both the patient and the examiner perceive the accent as foreign, (2) the accent represents a change from the patient’s accent before brain injury, (3) it is neurogenic, and (4) the patient does not speak additional languages (i.e., it is not polyglot aphasia, which shows different recovery of recent versus primary languages). Broca (1824–1880) introduced the term *aphemia*, which *refers to a* deficit in motor speech production or ‘speech loss.’ Marie (1907) used the term *anarthria*, which signifies the inability or difficulty in articulating speech. Monrad-Krohn (1947a) coined the term *dysprosody of speech* to describe what he identified as the disturbance of the ‘melody’ of spoken language, which includes alterations in syllable and word stress, rhythm, and pitch. Krohn emphasized that speech prosody is crucial for conveying meaning and emotion. He categorized organic dysprosody as a component of aphasia, with speech featuring a “foreign accent” as a type of dysprosody. The earlier terms were less precise or descriptive than the label foreign accent syndrome, which was ultimately adopted. Subsequently, the term ‘foreign accent syndrome’ was broadened to encompass psychogenic as well as neurogenic origins. Miller et al. (2006) noted that the perception of ‘foreignness’ appears to reside in the listener—who identifies various accents—rather than in the speaker, and may reflect the interplay of several speech changes, complicating the use of a specific descriptive term.

## In the beginning, initial case descriptions

As anticipated for a novel clinical syndrome, the contemporary record of acquired changes in accent begins with case descriptions, initially provided by Marie in 1907, followed by more detailed accounts by Pick in 1919, and likely most familiar to English-speaking readers, Monrad-Krohn in 1947.

Pierre Marie (1853–1940) trained under Broca and Charcot and initially aligned with his mentors in viewing separate receptive and expressive speech centers. However, by 1906, his perspective shifted, arguing that the left frontal region described by Broca was significant only for motor speech. He contended that deficits in language and intellect required lesions extending into the posterior language areas (Coutinho et al., 2021; Lecours et al., 1992). His analysis of clinical cases and a review of Leborgne’s preserved brain suggested that the lesion extended beyond Broca’s area, although it seemed more defined in Lelong (Coutinho et al., 2021). Marie challenged the classical localizationist perspective of Charcot, Lichthiem, and others, advocating for a more holistic conceptualization supported by Hughlings Jackson, Freud, and others (Tsapkini et al., 2008). Marie’s studies in 1906 sparked renewed discussion, making the discovery of patients exhibiting relatively pure speech issues without pronounced language issues (“anarthria”) crucial for Marie and his supporters.

Marie (1907) briefly described an individual with an altered accent, along with several other cases of anarthria. This individual had experienced a stroke that caused right hemiplegia and required 9 years to regain speech. However, he reportedly “has recovered language and can express his thoughts with all the necessary detail; he reads and writes very well (with his left hand); the inner language is not sensibly affected in him. However, there remain very noticeable remnants of his earlier anarthria, consisting of a rather pronounced Alsatian accent that he did not have before, as he is Parisian” (p. 864). Marie offered little additional description apart from speculating that the cause of the injury was a cerebral hemorrhage, attributing recovery to some regeneration of nerve fibers. The reported recovery of language, alongside the persistence of altered speech, aligns with Marie’s perspective of speech impairment without language impairment. It is worth noting that the focus was on speech rather than written expression, which seems a significant oversight from today’s viewpoint but may reflect the attitudes of a time before nearly universal adult literacy. The reported recovery of language alongside the persistence of altered speech aligns with Marie’s conception of speech impairment without language impairment.

Arnold Pick (19851–1924) was born in Moravia, which was part of the Austro-Hungarian Empire, a multilingual and multiethnic state where he primarily spoke German and Czech. He trained under Meynert and completed some training alongside Wernicke (Kertesz and Kalvach, 1996). He served as a professor of neuropathology and neuropsychiatry in Prague and had a wide range of interests, particularly in aphasia, especially agrammatical speech disorders (Kertesz and Kalvach, 1996). He remained active until 1921 and maintained correspondence with other leading neurologists, including Hughlings Jackson, Dejerine, and Pierre Marie; thus, he was undoubtedly aware of the localizationist/holistic controversy regarding language and aphasia, although he was not an active participant. This background shaped his description and formal assessment of a patient presenting with altered speech characteristics.

Pick (1919) provided the first detailed case description, contrasting with the single paragraph by Marie. This case involved a patient he assessed in 1914 at the invitation of a colleague. The patient, a Czech army medic, had suffered a stroke 2 years prior, resulting in unconsciousness, significant initial language impairment, and an inability to write despite no motor impairment. Although he showed overall good recovery and regained speech after 4 months, he continued to have unusual changes in speech characterized by a “Polish” accent. It was speculated that this reflected his service in either the Austrian-Polish or Russian-Polish army, but both the soldier and his wife indicated that this speech pattern developed after his stroke. Pick’s description was lengthy (12 journal pages) and detailed, as expected from his interest in language characterization. It was written in both German and Czech, mirroring the multilingual environment of the empire and the author’s background. Pick noted the differences in stress, altered vowel length, and ‘harshness’ between Polish and Czech speech styles. He provided a detailed transcript of his conversation with the patient, along with performance on tasks such as naming, comprehension, reading, writing, and sequencing. Pick identified agrammatic speech, appropriate comprehension, slowed performance on language tasks, significant difficulties with writing words and sentences, and notable amusia. He also mentioned having seen another patient with altered pronunciation recently, suggesting that this type of speech disorder was unrecognized rather than extremely rare. This case clearly indicated that, at least for this patient, there were mild language issues alongside speech changes, as well as some difficulties with written language.

G.H. Monrad-Krohn (1884–1964) provided the first case description in English. Born and educated in Norway, he also studied in Berlin and London, making visits to Paris, where he met the French proponents of the localizationist/holistic debates (Refsum, 1964). His interests included various language aspects and disruptions, reflexes (including those related to leprosy), and, crucial for our discussion, prosody beginning in 1947. His clinical approach started with a clear description of function, followed by identifying nervous system correlates and ultimately diagnosing the process and etiology, which had a significant impact on clinical training (Refsum, 1964).

In these initial English-language articles, Monrad-Krohn (1947a, 1947b) provided a detailed case study of Astrid L., who developed a German-sounding accent while recovering from a traumatic brain injury sustained when she was struck by bomb fragments, resulting in significant damage to her left frontal lobe. This case was thoroughly documented, benefiting from X-rays that illustrated a notable left frontotemporal parietal skull defect, and from inpatient care. She experienced nearly 4 days of unconsciousness, early right hemiplegia and aphasia, and suffered several seizures in the months following the injury. After 2 years, she demonstrated good motor recovery but retained a distinct “German or French-sounding” accent. The case presentation followed a structure similar to Pick’s, describing her physical functioning with brisk right-sided reflexes while displaying appropriate coordination and sensation. Language assessment and description were the primary focus, noting generally appropriate performance but some hesitations in speech and difficulties with written language. She showed mild agrammatism, and her melody of spoken Norwegian was noticeably altered when speaking multi-word phrases and sentences, exhibiting somewhat increased pitch variations but inconsistent presentation. In contrast to Pick’s case, which noted amusia, Monrad-Krohn indicated that Astrid L.’s musical abilities (rhythm, singing, and harmony) remained intact despite her altered speech. As the disconnection between intact musical abilities and altered speech melody became more apparent, he introduced the term ‘dysprosody’ to replace his earlier phrase ‘altered melody of speech’ in order to prevent confusion (Monrad-Krohn, 1947b). He noted that she continued to show improvement in some of her speech characteristics over time, which clearly indicated some practice in these skills. Ryalls and Reinvang (1985) were able to examine autopsy reports for this patient following her death in 1971. This is significant as it provided a detailed autopsy of one of the classic patients, which was not available for earlier cases. The autopsy showed the original lesion on the lateral side of the left frontal lobe, which had destroyed the larger part of the frontal lobe, measuring 9 cm anteroposterior and 6–7 cm in breadth. The lesion had destroyed Broca’s area and extended posteriorly near the speech center, providing some support for Marie’s position. The lesion extended into the left lateral ventricle, and both the left thalamus and basal ganglia exhibited significant atrophy. Additionally, a tumor was found in the right hemisphere, identified as a metastasis from bronchial carcinoma.

In all these cases, speech was absent for an extended period and gradually, albeit only partially, recovered. A contemporary perspective would emphasize brain plasticity and a dynamic lesion, with recovery reflecting both initial improvements from acute factors (edema, vascular disruption) and later factors such as incorporating nearby or functionally associated regions into active functional networks (Cramer, 2008).

Case reports continued throughout the 1960s, gradually identifying other pathologies that altered speech prosody and accent, such as brain tumors or strokes (Critchley, 1962). The inability to visualize brain lesions in living patients limited the available information, resulting in insufficient detail regarding specific brain structures beyond general laterality and lobes. These descriptions were provided by neurologists; it appears that specialists in language, including linguists or speech-language pathologists, were not engaged in defining these individual speech patterns or identifying common characteristics. This parallels other instances where neurology or psychology seeks to explain language features within their own fields (e.g., Friedrich, 2006). Table 1 provides a summary of the initial case descriptions and methodologies employed.

**Table 1:** Case details with presumed etiology.

Presumed etiology	Case	Year	Author	Discipline	Description and investigation	Description of “accent”
Stroke with right hemiplegia	Parisian man	1907	Pierre Marie (1907)	Neurology	Brief case description	Alsatian accent
Stroke, unconsciousness	Czech army medic	1919	Pick (1919)	Neurology	Detailed description, transcript of conversation, cognitive testing	Polish accent
Left hemisphere stroke with aphasia and weakness	65-year-old “new man with the foreign accent”	1961	Nielsen and McKeown	Neurology	Case description	Swedish accent
Stroke, left hemiplegia	E.L.	1962	Critchley	Neurology	Detailed description, Clinical examination	Welch accent
Traumatic left frontal brain injury	Astrid L.	1947	Monrad-Krohn	Neurology	Physical function, language assessment and description; X-ray*	“German or French” accent
Traumatic brain injury (multifocal), left hemiplegia	41-year-old man	1961	Nielsen and McKeown	Neurology	Case description	Unspecified “foreign accent”
Arteriovenous Malformation	27-year-old woman	1964	Whitty	Neurology	Clinical examination; electroencephalogram; carotid angiogram; skull radiographs	German accent
Psychogenic	M. E.	1962	Critchley	Neurology	Clinical examination	Welch accent
Psychogenic	37-year-old woman	1975	Darley	Speech Language	Clinical examination	German accent

^*^Autopsy, Ryalls and Reinvang (1985), showed lesion on the lateral side of the left frontal lobe.

Nielsen and McKeown (1961) described two cases. The first case involved a left hemisphere stroke in 1958, which resulted in aphasia and weakness, leading to dysarthria, dysprosody, apraxia, and agraphia, along with a noticeable “Swedish” accent. The second case was a man who sustained injuries in a motor vehicle accident; he exhibited spastic left hemiplegia, prolonged dysphagia, and multifocal brain injuries identified during burr-hole surgery, along with a slow recovery. After suffering from early dysarthria, he displayed a ‘foreign’ accent of unspecified nature. Nielsen and McKeown (1961) emphasized the difference between the dysarthria noted in these cases and the dysprosody associated with a lack of inflection (following Monrad-Krohn, 1947b). They observed that the perception of a foreign accent relied on the listener’s experience rather than on the distinct characteristics of the speaker. This study appears to be the first non-European research indicating that an acquired ‘accent’ could also be present in the US.

MacDonald Critchley (1900–1997), a prominent English neurologist with diverse interests in language and music (Joynt, 1998), documented the case of a 37-year-old female patient (E. L.) who experienced the onset of a “Welsh” accent following a sudden decline in her level of consciousness, accompanied by left-sided hemiplegia, expressive aphasia, and initial comprehension difficulties (Critchley, 1962). The patient exhibited a significant change in personality, primarily marked by increased disinhibition. Her speech was notably fluent, articulated “rapidly in a staccato yet melodic fashion” (p. 187). This case may represent the first documented instance of a foreign accent following an injury to the right hemisphere.

Whitty (1964) described a case of a 27-year-old woman who experienced right-sided weakness, sensory changes, and aphasia, along with gradual motor and sensory improvement. However, she exhibited altered speech characterized by a ‘German’ accent and some dysarthria. Investigations revealed a small angiomatous malformation in the left pre-Rolandic and suprasylvian regions, accompanied by an underlying clot measuring 8 × 4 × 4 cm. Following the excision, she showed good recovery, and within 6 weeks, her speech returned to normal.

While the loss of musical ability was observed in a specific case, it was not identified in others. However, changes in prosody were noted in case descriptions from the 1960s regarding “Swedish” and “Welsh” accents. Despite these observations, neurologists faced significant challenges. The aforementioned case descriptions were crucial in identifying potential sources of injury, related deficits, and the recovery timeline. It was observed that a ‘foreign-sounding’ accent emerged later in the recovery process after severe injuries. The early, broader language deficits mainly involved expressive challenges but could also include initial receptive difficulties along with motor impairments. However, there was a limited ability to pinpoint the affected brain regions in living patients, except through comparisons with findings from previously autopsied cases.

These classic cases involved patients with known lesions resulting from trauma, stroke, and related causes, featuring clear physical correlates that facilitated localization and had a distinct onset. Consequently, they presented little challenge to the diagnostician while illustrating that speech changes could manifest in several forms. However, a second group of case descriptions emerged in the 1960s and later, uncovering a new source for the emergence of foreign-sounding accents attributable to functional or psychogenic factors.

## Appearance of psychogenic etiology

Functional or psychogenic presentations have a long and complex history; the earliest written records can be traced back to ancient Egyptian and Mesopotamian texts (Jędrzejczak and Owczarek, 2012). The changing terminology (e.g., spirits, hysteria, dissociation, Charcot’s functional/dynamic lesions of the nervous system) reflects evolving etiological perspectives on supernatural, psychological, or integrative biopsychosocial conceptualizations (Perez et al., 2021; Pick et al., 2020). It was not until the 1960s that case reports of foreign accent syndrome identified a psychogenic etiology. The first widely recognized case, described by Critchley (1962), involved the sudden emergence of a regional Welsh accent in M. E., a 49-year-old English woman, following a car accident 2 years earlier, during which she sustained a compound skull fracture and briefly lost consciousness. M. E. was considered to be experiencing post-traumatic syndrome alongside “principally neurotic” symptoms of dysarthria (p. 185). Her so-called “Welsh accent,” which she apparently had never possessed before, was characterized as a “curiously slow, syllabic and hesitant way of speaking, with an excessive range of modulation” (p. 185), which did not entirely fit the context. Critchley (1962), in his introduction to the various cases, noted the unique British implications of regional accents and suggested that local accents could be looked down upon, while some foreign accents (e.g., French) might be viewed as quite pleasing (Critchley, 1962, p. 183–184). Thus, an acquired accent could affect a person’s perceived social status, depending on the accent and local prejudices.

Darley (1975), an American clinical scientist specializing in speech-language disorders, summarized several cases of foreign accent syndrome, including some of his own with presumed psychogenic origins. One case involved a 37-year-old woman described as a “neurotic type” (p. 56) who faced relationship issues with her husband. For 2–3 years, she experienced episodes of blacking out, weakness, and convulsions without any known neurological cause. “One day,” she began speaking with a German accent. Although she had been exposed to German through her grandmother, she had never learned to speak or understand it. Her foreign accent persisted for 2 days before disappearing. These episodes recurred once or twice more. After receiving counseling, her seizures improved, and the episodes ceased.

Interestingly, another case of foreign accent syndrome with a presumed psychogenic origin was not published until 2001, by Gurd et al. (2001), describing a transition from an English to a French accent. It remains unclear why there was a gap of approximately 25 years between publications on psychogenic foreign accent syndrome. One possibility is the lack of a unifying model for psychogenic foreign accent syndrome, unlike the classical model of aphasia, which assumes specific lesions for neurogenic etiologies (Geschwind, 1970). Other instances of foreign accent syndrome may have appeared earlier in the psychoanalytic literature without being indexed as foreign accent syndrome, thus evading typical search strategies. Breuer and Freud (1895) discussed functional speech disorders, particularly the well-known case of Anna O. Among her reported symptoms was a “functional disorganization” (p. 13) of her speech, in which, having lost the ability to use words in her own language, she painstakingly combined 4–5 languages to create a type of almost incoherent speech (Breuer and Freud, 1957).

## Interdisciplinary perspectives

Given the linguistic factors in these cases and the questions regarding how speech changes might be perceived as an accent instead of simple dysarthria, it is not surprising that linguists and speech-language pathologists have participated in this work. Darley (1975) provided several case descriptions, both with and without clear neurological involvement, identifying specific characteristics—such as persistent vowel stress—that would be uncommon in native English speakers. Darley’s presentation in this article and his textbook (Darley et al., 1975) raised awareness among speech therapists regarding these potential presentations, emphasizing careful observation and readiness for unexpected findings. Such a call to action could increase the likelihood of identifying and reporting these unusual presentations.

Whitaker (1982), in a chapter on various disruptions to speech pathways and their interpretation, noted that cortical lesions could alter accents (pp. 195–206). He described how altered articulation and specific speech characteristics could create a perceived foreign accent and provided the first recorded instance of the term “foreign accent syndrome,” along with its four main characteristics. The chapter offered a linguistic description of a new case, similar to earlier ones. This study lamented the difficulties of case studies, where papers either provided solid anatomical data but lacked strong language characterization or the reverse, depending on the researchers’ backgrounds. This concern appears to have influenced subsequent reports, which more consistently integrate both linguistic descriptions and imaging findings, yielding greater detail and identifying both common and rare characteristics. This broader perspective appears to be a fitting response after basic descriptions were made and the concept of a psychogenic presentation was considered, as distinguishing between causes could have practical implications for education and suitable treatment.

## The introduction of technology

While case studies have been beneficial in identifying the phenomenon and certain related characteristics, they do not provide information regarding specific anatomical regions or functions. The examination of living patients has been essential for characterizing language; however, it hinders anatomical characterization unless the skull vault is surgically opened, as evidenced in the cases documented by Nielsen and McKeown (1961) and Whitty (1964). Structural brain imaging has played a crucial role in identifying lesions in individuals with traumatic injuries, and functional imaging has become increasingly important for delineating regional correlates of function from the 1980s to the present.

## Structural imaging

Structural imaging for patients with language disorders has evolved gradually, as the skull vault restricts the detail visible in plain X-rays, presenting a challenge that also affects other central nervous system disorders. Imaging of brain morphology awaited the advent of computerized tomography (CT). Noel et al. (1977) conducted one of the earliest CT studies on aphasic patients, while Mazzocchi and Vignolo (1979) compared imaging results with an aphasia battery in 90 patients. They found, as expected, that lesions affecting comprehension but allowing for fluent production were primarily located in the posterior regions, while patients with nonfluent aphasia predominantly exhibited frontal lesions, which generally (though not always) corresponded with classical neurological descriptions. Studies of patients with dysarthria (e.g., Arboix et al., 1990), on the other hand, typically showed lesions in classical motor pathway areas, such as the cerebellar and basal ganglia regions, rather than in cortical regions.

Schiff et al. (1983) examined four patients with aphemia (dysarthria without aphasia) who had CT-identified lesions in the left speech motor regions or associated subcortical areas. Some examiners reported that one patient, who was bilingual in Portuguese and English, spoke with a “Chinese” accent. This patient had exhibited transient right hemiparesis and experienced mild aphasia for 3 weeks following initial muteness. Imaging revealed a lesion in the lower half of the precentral gyrus and the underlying white matter.

Graff-Radford et al. (1986) reported on a 56-year-old female patient who presented with transcortical motor aphasia, initial dysarthria, limited speech output, and agraphia, all while showing a normal sensory examination. CT scans revealed a left-sided infarction in Brodmann’s area 6, along with associated deep white matter changes. Shortly after her discharge, acquaintances noted they perceived a foreign accent in her speech. Family and friends described her speech as resembling Nordic accents; however, those familiar with Scandinavian languages did not share this view. Furthermore, several individuals found it challenging to discern the emotional intent conveyed through her speech. She demonstrated appropriate visual perceptual skills and fluent language with infrequent paraphasic errors, although characterized by numerous pauses. Her spontaneous writing and dictation were completed with slowness yet accuracy, while her reading comprehension exhibited average accuracy at an extremely slow pace. Additionally, she displayed appropriate prosodic recognition, although an analysis of her speech quality indicated slower articulation, frequent pauses, elongation of certain words, and changes in her speech’s fundamental frequency. Notably, she showed several vowel shifts that corresponded with a more tense speech posture.

Blumstein et al. (1987) conducted a detailed case study of a 62-year-old woman who suffered a stroke in the left hemisphere, resulting in weakness on her right side and some diminished sensation. Within 2 weeks, her language performance was generally adequate, exhibiting somewhat slowed output, mild articulation abnormalities, and an altered melodic line and intonation. She was capable of producing lengthy, complex sentences and demonstrated adequate performance on neuropsychological assessments, showing some weakness in complex calculations but no signs of Gerstmann syndrome or memory impairment. However, her speech output was distinctly unusual; listeners agreed that she had a foreign accent despite differing opinions on the nature of the accent (e.g., Slavic, French, or Scandinavian). She displayed an altered speech melody (with equally stressed syllables and consistent rising intonation), modified consonant production, and a lack of positional phoneme changes. Many aspects of her speech aligned with her pre-injury expectations, although other characteristics—such as altered stop voicing and changed prosody contours—did not conform to what was anticipated based on her pre-injury recordings. This indicated an overall change in prosody and speech rhythm. The perception of a foreign accent was supported by near-normal lexical and grammatical features, along with the absence of dysarthria characteristics. CT scans revealed lesions in the left white matter beneath the facial sensory and motor cortex.

## Functional imaging

Structural imaging was somewhat useful in confirming that CT or MRI imaging broadly supported the classical localization of language presentation, with some individual variability. However, for patients with functional (or psychogenic) foreign accent syndrome, structural imaging provides little information, although it may indicate that a functional cause is more likely. In contrast, functional imaging proves more beneficial, particularly in suggesting mechanisms of symptom presentation. Unfortunately, for our purposes, the techniques of functional imaging (e.g., SPECT or fMRI) do not appear to have been applied to this population before 2000, with the earliest study (Fridriksson et al., 2005) conducted in 2005. This study examined a 45-year-old man who suffered a left subcortical ischemic stroke, initially presenting with facial droop and markedly slurred speech, which resolved within a few days. He was able to return to work but continued to speak with an altered accent. Six weeks post-stroke, he performed within normal limits on tests of language function, oral apraxia, executive functioning, and a general cognitive screen. Structural imaging showed a small infarct in the left putamen, while tractography indicated that projections between the internal capsule and corona radiata were largely spared. fMRI during a picture naming task revealed similar areas of activation as healthy controls, involving the superior temporal and inferior frontal cortex (corresponding to the classical Wernicke’s and Broca’s areas), along with regions related to facial and object recognition, consistent with those of the controls. Additionally, he exhibited increased activation in the left central sulcus and ventral angular gyrus. The authors suggested that this pattern of activity could indicate that putamen damage led to increased cortical motor processing as a compensatory mechanism.

Mariën et al. (2006) conducted a 3-year follow-up case study that examined a 53-year-old right-handed native speaker of Dutch who developed foreign accent syndrome (FAS) 3 weeks after experiencing a left fronto-parietal stroke, with initial a brief loss of speech. The patient’s FAS resolved 3 years post-stroke, although she displayed residual very mild apraxia of speech. A SPECT scan taken 33 days after the onset of neurological symptoms revealed disruption in the left frontal motor cortex, along with relative hypoperfusion noted in the thalamus, striatum, and left anterior temporal region, with corresponding hypoperfusion observed in the contralateral right cerebellum. A follow-up SPECT scan conducted after the remission of FAS showed normalization of the cerebellum, with only minor improvements noted in the supratentorial brain regions, specifically the thalamus, striatum, and left anterior temporal region. The authors concluded that the cerebellum may play a role in disorders associated with motor speech planning, including FAS.

Poulin et al. (2007) presented a case study of FG, a bipolar patient who experienced a sudden onset of FAS and agrammatism. An FDG-PET scan revealed diffuse bilateral hypometabolism in the frontal, parietal, and temporal lobes, along with a focal deficit in the anterior left insula and anterior temporal cortex, with prominence of the sylvian sulcus. Poulin concluded that the significant hypometabolism in the left insula and anterior temporal cortices was likely not attributable to the patient’s bipolar disorder or a neurodegenerative disorder but rather was related to his language symptoms.

Similar to other disorders, imaging techniques have evolved from merely identifying specific lesion locations to examining functional networks. This shift has become increasingly evident, as reviews indicate a wide variety of lesion locations, suggesting that there is no single critical site, or even lobe, that consistently produces FAS symptoms (e.g., Higashiyama et al., 2021). Early work by Fridriksson et al. (2005) exemplified this evolution, while contemporary studies have employed functional connectivity analyses to explore activation patterns in patients with psychogenic or functional origins. Dadario et al. (2023) conducted functional imaging studies on three patients recruited from two different countries, revealing the absence of structural lesions linked to altered speech. However, they noted overlapping activation patterns in regions associated with internal language processing, sensorimotor networks, and deep subcortical structures, particularly in patients 2 and 3 of their series. This field remains a dynamic area of research. Furthermore, the findings of this study highlight a recent trend toward multisite and even multicountry collaborations in the examination of rare conditions, moving away from the traditional reliance on single specialized clinics to gather a limited number of patients sharing a common diagnosis.

## Discussion

It is evident that the term “foreign accent syndrome” is somewhat misleading, as it does not accurately represent a syndrome; rather, it reflects a specific aspect of altered speech due to neurological or functional factors influencing prosody. Unlike a focal neurological syndrome, lesions have been identified across multiple brain regions. For instance, Higashiyama et al. (2021) conducted a review of imaging studies involving numerous cases, discovering lesions in various brain areas presumably associated with the symptoms, including the left frontal motor and premotor regions, basal ganglia/corona radiata, right hemisphere areas, brainstem, and cerebellum. This diversity of affected brain regions highlights the idea that multiple aspects of language changes can lead to subtly altered speech, further emphasizing the extensive brain regions and networks involved in the production of speech.

Examining the historical literature reveals an increased description of cases, along with a gradual improvement in detail and terminology that more effectively identifies the key features. Aphemia and anarthria are unfortunately nonspecific, while dysprosody of speech, although somewhat more specific, obscures the fundamental characteristics of this presentation. Although Pick’s (1919) description noted amusia, and it is superficially tempting to link altered prosody to some level of right hemisphere dysfunction, Monrad-Krohn’s careful examination and subsequent shift from using the *melody of speech* to *dysprosody of speech* emphasized that this was not a generalized dysfunction of pitch or tone production. Foreign accent syndrome, or acquired foreign accent, emphasizes the key feature for both the listener and often the patient, highlighting the specific nature of the speech alteration.

Beginning in the 1980s, the confirmation of lesions through structural imaging and the increased involvement of specialists beyond the field of neurology—including neuropsychologists, speech-language pathologists, and linguists—has facilitated the integration of models related to cognitive and language functions, thereby shedding light on the underlying processes (e.g., Moen, 2000; Scott et al., 2006). These methodologies have proven particularly valuable in cases where a focal neurological lesion has been identified. Nevertheless, functional and connectivity imaging has had a significant impact in this area. The wide variety of lesion loci indicates that there is no single critical area responsible for these symptoms. Furthermore, as the functional presentations make structural imaging largely uninformative, functional imaging that identifies specific sites showing increased or decreased metabolic activity, along with changes in functional connectivity, provides a better understanding of the specific foundations of this presentation, incorporating the broader networks that govern language and speech motor control. Through repeated imaging, it becomes possible to detect changes in activation and functional networks associated with symptom amelioration or deterioration, as shown by Mariën et al. (2006), who reported a normalization in contralateral cerebellar activation alongside reduced FAS, despite no noticeable changes in cortical regions.

This current emphasis on networks involving multiple brain regions (both cortical and subcortical), functioning in a modular and coordinated manner rather than in a sequential pathway from conceptualization to speech or writing, aligns with some of Marie’s ideas from 1906 and beyond. The extensive destruction of brain tissue noted in Astrid L, for example (Ryalls and Reinvang, 1985), suggests that Broca’s area played a significant role, but subsequent adaptation and resumption of (altered) speech were possible. The absence of a ‘critical lesion area’ (although the HCP premotor area 55b has been proposed as a common site for lesions or dysfunction; Dadario et al., 2023) indicates that this cannot be seen as a dysfunction of a single aspect of language or speech. Instead, it may arise from dysfunction at multiple points within the speech control network. A functional network conceptualization can improve our understanding of the recovery of function, particularly when lesions and associated brain metabolism may remain unchanged or when attempting to grasp the variable pace of recovery. As noted by Tsapkini et al. (2008), there is ongoing controversy regarding the role of speech output areas and their connection to broader intellectual and language functioning. Detailed analyses of speech and language have shown that patients can recover language skills despite altered speech (e.g., Fridriksson et al., 2005), and they have exhibited subtle language deficits following initial severe injuries that Marie overlooked in his original presentation (as described in Pick and Monrad-Krohn’s presentations).

However, notable gaps exist in the historical overview we have presented. First, although a functional or conversion cause for foreign accent has been reported multiple times over the past 50 years, especially since 2001, we have not been able to locate earlier accounts despite the clear interest in this topic from a psychoanalytic perspective. This gap may simply reflect variations in terminology, as psychoanalysts may not use terms like aphemia or anarthria. A thorough review of existing literature for earlier case descriptions could provide valuable insights. A second significant gap lies in the absence of linguistic description and analysis, or the involvement of speech-language pathologists for treatment, before Darley’s exposition in 1975, even though speech remediation and linguistic analyses of aphasic speech have been established for many decades, as noted by Eling and Whitaker (2022). Fortunately, linguists have increasingly engaged in this area and have offered conceptual frameworks since the early 1980s, leading to more precise descriptions.

This topic has been discussed in the literature for over a century. However, much of the research on mechanisms and networks, as well as the elaboration of psychogenic foreign accent acquisition, has occurred since 2000.

In cases of psychogenic FAS, no unifying cause has been identified and is not expected. A review by Keulen et al. (2016) noted diagnoses including conversion disorder (functional neurological disorder), bipolar disorder, schizophrenia, and obsessive-compulsive disorder observed in case reports, with the etiology remaining speculative. Functional imaging of one case indicated bilateral diffuse frontal, parietal, and temporal hypometabolism, although it was not at a level sufficient to explain the language changes alone. Corroborating findings with additional cases and comparing functional imaging from other functional cases or related disorders (such as bipolar disorder or OCD) will be crucial to determine if there is a distinctive pattern. However, Dadario et al. (2023) found varying patterns of functional coactivation in three patients, with two showing substantially similar patterns and the third presenting a distinct pattern.

This presentation continues to fascinate the general public, although popular perceptions are often inaccurate. The recent trend of including patients in research and public presentations may improve understanding and explanations for both the public and healthcare providers who encounter this presentation (Miller et al., 2011). This is beyond the scope of this article, but increasing engagement suggests that this phenomenon, although uncommon, is not exceedingly rare (Ryalls and Miller, 2014). Media portrayals, such as those in videos and news broadcasts, may have increased the prominence and prevalence of functional foreign accent presentations in the last two decades.

The literature has shown increased sophistication and elaboration of models that explain both normal and altered speech. The progression from earlier strict localizationist views (e.g., based on Lichtheim or Charcot’s models) has shown that altered accents can occur without significant language changes or as a late stage of recovery from early severe language issues. This serves as an important counter-example of speech alteration as a form of dysprosody, while increasingly detailed examinations have indicated that there were some residual language deficits. More contemporary descriptions (e.g., Higashiyama et al., 2021; Moen, 2000) suggest altered phonetic settings and vocal ranges, changes in the control of speech motor behavior, cognitive shifts that require more effortful control of speech, or the utilization of less effective motor strategies to scale and blend articulatory movements.

Finally, Whitaker’s (1982) observation that single discipline approaches are less fruitful than collaboration remains true, for this concern as for many others. Integration of localization, linguistic analysis, associated signs and symptoms, personal experiences, and treatment modalities is paramount to fostering a deeper understanding of the individual and enhancing treatment efficacy. This principle is applicable across a diverse array of disorders exhibiting neurological, cognitive, psychological, and systemic characteristics, encompassing conditions such as systemic lupus erythematosus or, more recently, infections caused by COVID-19. Investigating how an integrated assessment and diagnostic approach has contributed to the advancement of understanding and treatment of specific disorders may prove beneficial for practitioners as they collaborate to address newly identified challenges.
